# Study within a trial (SWAT) protocol. Participants' perspectives and preferences on clinical trial result dissemination: The TRUST Thyroid Trial experience

**DOI:** 10.1016/j.conctc.2017.07.001

**Published:** 2017-07-04

**Authors:** Emmy Racine, Caroline Hurley, Aoife Cheung, Carol Sinnott, Karen Matvienko-Sikar, William H. Smithson, Patricia M. Kearney

**Affiliations:** a Department of Epidemiology and Public Health, University College Cork, Ireland; b Department of General Practice, University College Cork, Ireland

**Keywords:** Public and patient involvement, PPI, Study within a trial, SWAT, Clinical trial result dissemination, End of trial results, Trial results

## Abstract

**Introduction:**

Dissemination of results of randomised controlled trials is traditionally limited to academic and professional groups rather than clinical trial participants. While there is increasing consensus that results should be communicated to trial participants, there is a lack of evidence on the most appropriate methods of dissemination. This study within a trial (SWAT) aims to address this gap by using a public and patient involvement (PPI) approach to identify, develop and evaluate a patient-preferred method of receiving trial results of the Thyroid Hormone Replacement for Subclinical Hypothyroidism Trial (TRUST).

**Methods:**

An experimental (intervention) study will be conducted using mixed methods to inform the development of and evaluation of a patient-preferred method of communication of trial results. The study will involve three consecutive phases. In the first phase, focus groups of trial participants will be conducted to identify a patient-preferred method of receiving trial results. The method will be developed and then assessed and refined by a patient and public expert group. In the second phase participants will be randomly assigned to the intervention (patient-preferred method) and comparison groups (standard dissemination method as developed by the lead study site in Glasgow, Scotland). In the third phase, a quantitative questionnaire will be used to measure and compare patient understanding of trial results between the two groups.

**Discussion:**

This protocol provides a template for other trialists who wish to enhance patient and public involvement and additionally, will provide empirical evidence on how trialists should best disseminate study results to their participants.

## 1 Background

Dissemination of trial results has traditionally been limited to three channels: scientific meetings and peer reviewed publications and texts; lay media; and groups and organizations with a particular health interest [1]. However, the most recent iterative of the EU Clinical Trials Regulation 536/2014 (Article 37) requires sponsors to provide summary results of clinical trials in a format understandable to laypersons [2]. Therefore, disclosure of study results to trial participants is now a mandatory fourth channel of dissemination. While it is desirable that results are shared with study participants, there is a lack of evidence on the most appropriate methods of sharing research findings with these important stakeholders [1]. Since the findings exist in a context of scientific exchange and debate, researchers need to ensure that the information presented to participants meets their needs [1].

The move to disseminate trial results to trial participants had occurred contemporaneously with the expanding role of Patient and Public Involvement (PPI) in research. The Involve (UK) definition of PPI is widely used and defines public involvement in research as research being carried out ‘with’ or ‘by’ members of the public rather than ‘to’, ‘about’ or ‘for’ them [3]. The goal of PPI is to achieve a true partnership between public/patients and researchers, leading to improved research quality, relevance and outcomes. While PPI is gathering considerable momentum internationally, limited attention has been placed on measuring the impact of PPI [5], [6]. Research in this area has often found to be of poor quality and there have been difficulties in identifying the exact contribution patient involvement has on overall patient care [7]. For these reasons a clear need has been established to measure the impact of PPI in a valid, reliable and responsive way [5].

This study within a trial (SWAT) aims to address the lack of evidence on the most appropriate methods of sharing research findings with trial participants by using a PPI approach to identify, develop and evaluate a patient-preferred method of receiving trial results within the Thyroid Hormone Replacement for Subclinical Hypothyroidism Trial (TRUST).

The TRUST clinical trial is a multi-centre, double blind, placebo controlled, phase III clinical trial testing the efficacy of thyroxine replacement in subclinical hypothyroidism in older community dwelling adults. The study sites for the trial are the University of Glasgow, Scotland (lead site); Leiden Academy on Vitality and Ageing, The Netherlands; Leiden University Medical Centre, The Netherlands; University of Berne, Switzerland; and University College Cork, Ireland. This collaborative academic study recruited 738 participants with subclinical hypothyroidism (SCH) over a three-and-a-half-year period from 2013 to 2017 [4]. A total of 115 participants were recruited in Ireland. The TRUST Trial completed recruitment in November 2016 and the results will be reported in Spring 2017.

## 2 Methods

**Fig. 1:**
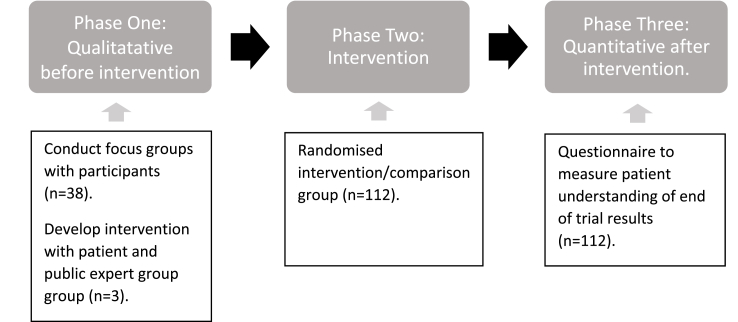
Study Design.

**Study Design:** This study will adopt an experimental (intervention) design using mixed methods to inform the development of and evaluation of a patient-preferred method of communication of trial results. The design consists of three distinct phases (see Fig. 1).

**Setting:** The study will be conducted at the Irish TRUST site. The hub centre for the Irish site was located at the Mercy University Hospital, Cork with five satellite sites located at Waterford University Hospital, Bantry General Hospital, Kerry General Hospital, St John's Hospital Limerick and Vista Primary Care Centre, Naas. A total of 115 participants were recruited from these Irish sites and randomised to the TRUST Trial.

**Population:** As this is a SWAT within the TRUST trial, the study sample is determined by the trial. This study will consist of all TRUST participants recruited in the Irish site (n = 115).

### 2.1 Phase one: qualitative data collection

**Aim:** To use a PPI approach to explore participants' perspectives and preferences of result dissemination. This information will identify and guide the development of the PPI informed result dissemination method.

**Design:** Qualitative focus group study.

Method:

#### 2.1.1 Focus groups

Three semi-structured focus groups will be conducted with four to eight TRUST participants per focus group. Participants for this phase will be determined based on the geographical feasibility of carrying out the focus groups. Cork is the main site for TRUST in Ireland therefore all Cork-based patients (n = 38) will be contacted via letter and invited to participate in one of three focus groups in the Cork TRUST site. The sessions will be led by an independent qualitative researcher. A topic guide will be used to guide the focus groups. The Consensus-Oriented-Decision-Making (CODM) model will be used in order to guide the group to reach a consensus. The CODM model steps [8], [9] include:

1 Framing the topic
2 Open discussion
3 Identifying underlying concerns
4 Collaborative proposal building
5 Choosing a direction
6 Synthesizing a final proposal
7 Closure.

**Analysis:** Focus group recordings will be transcribed verbatim and entered into NVivo Version 11 for thematic analysis. Braun and Clarke [9] guidelines will be followed when thematic analysis is being performed. Identified themes will be used to model a patient-preferred method for the dissemination of results.

#### 2.1.2 Patient and public expert group

A patient and public expert group will be established to assess the content on and test the validity of the proposed trial result dissemination method. This group will consist of three to five TRUST participants recruited from the Cork site. A series of consultations will take place with this group in order to iteratively refine the format of the patient-preferred dissemination method.

**Outcome:** The findings from this phase will be used to identify and develop the patient-preferred end-of-trial result dissemination method.

### 2.2 Phase two: intervention

**Aim:** To disseminate the results of the TRUST Trial to TRUST participants.

**Design:** A prospective, randomised, single-blind, parallel-group intervention group trial.

**Method:** Results will be disseminated to participants in Ireland after the final results of the trial are published in April 2017. Simple random allocation will be used to randomise participants to the intervention or comparison group. Participants from the patient and public expert group (n = 3) will not be randomised as they will have already reviewed the content of the intervention method prior to randomisation. The intervention group will receive the PPI informed dissemination method and the comparison group will receive the standard dissemination method developed by the lead study site in Glasgow, Scotland. Participants will be blinded to the result method they will receive. One member of the research team will be un-blinded in order to perform the randomisation and distribute the results of the trial.

**Outcome:** All TRUST participants will receive the results of the TRUST Thyroid Trial.

### 2.3 Phase three: quantitative data collection

**Aim:** To measure the impact of PPI on patient understanding of end-of-trial results.

**Design:** Quantitative questionnaire evaluation of the intervention.

**Method**: A questionnaire will be developed and validated to measure patient understanding of the end-of trial results. The questionnaire will be developed in consultation with experts in the area of subclinical hypothyroidism and scale development. It is expected that the questionnaire will contain no more than 10 items which will be measured on a LIKERT scale. While there is debate in the literature regarding the minimum required sample size for exploratory factor analysis, it has been established that a ratio of 10 participants per variable is appropriate to produce solutions that are accurate representations of the population parameters [10]. Questionnaire items will be developed by adapting existing questionnaires and new items will be generated where necessary through consultation with the content and scale development experts and the patient and public expert group. Prior to administering the full questionnaire, it will be reviewed by the patient and public expert group to assess content and face validity. Any suggestions and changes made by this group will be incorporated into the final questionnaire. The questionnaire will be sent to the intervention and comparison groups one week after they have received the results of the trial.

**Analysis:** Completed questionnaires will be entered into SPSS software. The psychometric properties and construct validity of the questionnaire will be examined with exploratory factor analysis, using principal component analysis (PCA). PCA is a useful exploratory factor analytic tool that can be incorporated into the development and refinement of new scales [11]. In this case, PCA will allow for the maximum amount of variance in patient understanding to be explained by the smallest number of constructs. Internal consistency of the questionnaire will be investigated using Cronbach's alpha coefficient. Where possible, participant responses will be analysed using descriptive and inferential (Chi-square test) statistics. The researcher carrying out the input and analysis of this data will be blinded to the participants' allocation status. Subgroup analysis will also be conducted to measure how patient understanding is impacted by age, gender, education status and study site attended.

**Outcome:** The primary outcome will be difference in levels of patient understanding between the intervention and comparison groups. This will measure the impact of PPI on patient understanding of end of trial results.

## 3 Discussion

The importance of engaging patients in the design and conduct of clinical trials is increasingly recognised [1]. This protocol sets out a rigorous approach to engaging patients in decisions about how best to communicate trial results with trial participants. It will provide a useful template for other clinical trialists who wish to enhance patient and public involvement and additionally, will provide empirical evidence on how trialists should best disseminate study results to their participants.

## Ethics approval

The research was approved in Ireland by the Clinical Research Ethics Committee of the Cork Teaching Hospitals, UCC, Ref ECM 4 (t).

## Funding statement

This work was supported by Health Research Board -Trials Methodology Research Network (HRB-TMRN). The TRUST Trial is funded under the EU's FP7 programme.

## Competing interests

The authors declare that they have no competing interests.

## List of abbreviations

SWAT: Study Within a Trial
TRUST: Thyroid Hormone Replacement for Subclinical Hypo-Thyroidism Trial
PPI: Public Patient Involvement
SCH: Subclinical Hypothyroidism
